# Factors Predicting the Coronavirus Disease 2019 Preventive Behaviors of Older Adults: A Cross-Sectional Study in Bangkok, Thailand

**DOI:** 10.3390/ijerph191610361

**Published:** 2022-08-19

**Authors:** Chunphen Upake, Sutham Nanthamongkolchai, Pimsurang Taechaboonsermsak, Korravarn Yodmai, Wanich Suksatan

**Affiliations:** 1 Department of Family Health, Faculty of Public Health, Mahidol University, Bangkok 10400, Thailand; 2 Faculty of Nursing, HRH Princess Chulabhorn College of Medical Science, Chulabhorn Royal Academy, Bangkok 10210, Thailand

**Keywords:** older adults, COVID-19, health behaviors, prevention, health promotion

## Abstract

The coronavirus disease 2019 (COVID-19) pandemic has affected the health behaviors of older adults. Thus, the factors predicting the COVID-19 preventive behaviors of older adults during the COVID-19 outbreak should be examined. Therefore, this study aimed to assess the COVID-19 preventive behaviors of older adults and explore the factors predicting these. A cross-sectional study was performed with 400 older adults who were selected using the cluster sampling technique. The associations of all variables in preventing COVID-19 infection with COVID-19 preventive behaviors were examined using stepwise multiple regression. The study results revealed that 70.8% of the study participants had high levels of COVID-19 preventive behaviors. Among these, self-efficacy in preventing COVID-19 infection (β = 0.224) showed the highest ability to predict COVID-19 preventive behaviors, followed by COVID-19 response efficacy (β = 0.171), knowledge about COVID-19 (β = 0.110), and gender (β = −0.102). Older adults adopted protective behaviors at the beginning of the COVID-19 pandemic. The predictors of these behaviors should be considered while designing and developing appropriate COVID-19 preventive behavior interventions, aimed at inducing behavioral modifications to reduce further infection with and spread of COVID-19.

## 1. Introduction

Coronavirus disease 2019 (COVID-19), which is caused by severe acute respiratory syndrome coronavirus 2 [1], is a new communicable respiratory disease that was first discovered in December 2019 in Wuhan, Hubei, China, and spread to the rest of the world [2]. As of July 11, 2022 [2], there have been 552,504,626 confirmed cases of COVID-19 worldwide, and a total of 6,347,816 people (2.07%) have died from the disease. The United States has had the highest number of COVID-19 infections and deaths, with a total of 65,178,846 infections and 1,593,940 deaths [3]. In Thailand, there have been 4,546,854 confirmed cases of COVID-19 and 30,859 deaths [4]. The COVID-19 pandemic continues to threaten the health of people worldwide [5,6], as the highly communicable disease can spread rapidly and infect large numbers of people [7,8].

COVID-19 outbreaks continue to occur in many areas of Thailand, with rising numbers of infected patients, particularly among the older adult population [8,9]. A study conducted among older adults concerning their practices for preventing COVID-19 found the highest mean scores for mask wearing, eating, and personal beliefs [10], and the lowest mean scores for behavior and handwashing [10]. Another study revealed that older adults had a good to moderate preparedness level for COVID-19 preventive behaviors and control (76.5%), while the proportion with poor COVID-19 preventive behaviors was 23.5%. Regarding the older adults’ knowledge about COVID-19 prevention and control, it was found that the majority of them (78.4%) had poor knowledge, perhaps because 84.4% of them were illiterate [11]. Good COVID-19 preventive behaviors and knowledge about COVID-19 are important for every age group, but especially for older adults (60–69 years), who are at greater risk of infection than younger people because many of them continue to perform activities in person, travel to work, and meet people [12,13].

Despite the extensive efforts to contain the COVID-19 pandemic, the number of older adults who have been infected with COVID-19 has increased exponentially in over 100 countries, resulting in millions of deaths globally. Older people are more likely to develop severe COVID-19 than younger people [14]. In critical situations, older adults may require hospital admission, critical care, or a ventilator to help them breathe [14]. Therefore, individual behavior has a substantial impact on disease prevention and control. Regarding the COVID-19 pandemic, inadequate knowledge about COVID-19 can have negative repercussions, whereas having adequate knowledge about COVID-19 can help prevent infection and the further spread of the disease.

Self-efficacy refers to one’s belief and confidence in one’s ability to achieve behavioral goals in a particular area [15]. According to Bandura et al. [15], individual cognition can influence behavioral regulation, and self-efficacy (a cognitive factor) is a crucial psychological motivator for maintaining individual self-regulation and health behaviors. Thus, we became interested in studying COVID-19 preventive behaviors based on the protection motivation theory [16] to develop a conceptual framework for promoting COVID-19 prevention among older people, emphasizing that older people must be aware of the fact that improper practices will increase their COVID-19 risk. With appropriate awareness, older people can improve their behaviors, become motivated to overcome barriers, and accept preventive behaviors as part of their daily lives. Therefore, the aim of this study was to investigate the COVID-19 preventive behaviors of older adults and the factors that can predict COVID-19 preventive behaviors among older adults in Bangkok, Thailand, using our findings to prepare health promotion guidelines for older people and help them improve their preventive behavior capabilities in preparation for future health crises.

## 2. Materials and Methods

### 2.1. Study Design and Sampling

A cross-sectional predictive research design was employed. The Strengthening the Reporting of Observational Studies in Epidemiology statement for cross-sectional studies was used to report the conduct and findings of this study [17]. The data were collected through self-report questionnaires distributed to older people in the Thung Phaya Thai and Nuan Chan districts in Bangkok, Thailand. The sample size of the study was determined based on Daniel’s [18] formula; thus, the anticipated sample size of the study was 400. Cluster random sampling was used to recruit potential participants. The inclusion criteria were (1) adults aged 60–69 years; (2) living in Bangkok, Thailand, during the COVID-19 pandemic; (3) no history of cognitive dysfunction, Alzheimer’s disease, or psychiatric illness; and (4) able to communicate in the Thai language. Those who felt uncomfortable or had any health-related symptoms (e.g., dyspnea, dizziness) and were unable to participate in the study for other reasons were excluded from the study. We collected the study data from 400 older adults in Bangkok Metropolitan, Thailand, using a self-reported questionnaire distributed from 14 November to 14 December 2021.

### 2.2. Research Instruments

Seven questionnaires, one each on knowledge about COVID-19, perceived COVID-19 risk, perceived COVID-19 severity, COVID-19 response efficacy, self-efficacy in preventing COVID-19 infection, and COVID-19 preventive behaviors, were developed by the principal investigator (PI) based on a literature review and were used for data collection in this study. The contents of all the questionnaires were validated by a public health expert, a professional nurse, and a gerontologist. All the questionnaires were then tested for reliability in 35 older adults with characteristics, such as those in the main study population, to confirm the reliability of the questionnaires before collecting data in this study.

### 2.3. Knowledge about COVID-19

The questionnaire on knowledge about COVID-19 consisted of 15 items, each falling under one of the following four subscales: (1) disease cause; (2) signs and symptoms; (3) incubation period; and (4) transmission route. It used rating scales from 1 to 0, where a score of 1 is given to the correct answer and 0 to the incorrect answer. The total score ranged from 0 to 15, and mean scores were categorized into three levels according to Bloom [19]—low level (15–59), moderate level (60–79), and high level (80–100)—with a higher score indicating greater knowledge about COVID-19. The questionnaire was analyzed for internal consistency reliability using the Kuder–Richardson method, and it obtained a score of 0.76, which is considered to represent a reasonable level of internal consistency reliability.

### 2.4. Perceived COVID-19 Risk

The questionnaire on perceived COVID-19 risk consisted of eight items, each rated on a 5-point scale, with: 1 = “strongly disagree” and 5 = “strongly agree.” The total score ranged from 840, and mean scores were categorized into three levels according to Bloom [19]—low level (15–59), moderate level (60–79), and high level (80–100)—with a higher score indicating a higher perceived COVID-19 risk. The content validity index (CVI) of perceived COVID-19 risk was 0.87. The Cronbach’s alpha coefficient of the questionnaire was 0.75, indicating good internal consistency reliability.

### 2.5. Perceived COVID-19 Severity

The questionnaire on perceived COVID-19 severity consisted of nine items, each rated based on a 5-point scale: 1 = “strongly disagree” and 5 = “strongly agree.” The total score ranged from 9 to 45, and mean scores were categorized into three levels according to Bloom [19]: low level (15–59), moderate level (60–79), and high level (80–100), with a higher score indicating higher perceived COVID-19 severity. The CVI of perceived COVID-19 severity was 0.88. The Cronbach’s alpha coefficient of the questionnaire was 0.74, indicating good internal consistency reliability.

### 2.6. COVID-19 Response Efficacy

The questionnaire on COVID-19 response efficacy consisted of nine items, each rated based on a 5-point scale: 1 = “strongly disagree” and 5 = “strongly agree.” The total score ranged from 9 to 45, and mean scores were categorized into three levels according to Bloom [19]—low level (15–59), moderate level (60–79), and high level (80–100)—with a higher score indicating higher COVID-19 response efficacy. The CVI of COVID-19 response efficacy was 0.77. The Cronbach’s alpha coefficient of the questionnaire was 0.81, indicating good internal consistency reliability.

### 2.7. Self-Efficacy in Preventing COVID-19 Infection

The questionnaire on self-efficacy in preventing COVID-19 infection consisted of nine items, each rated based on a 5-point scale: 1 = “strongly disagree” and 5 = “strongly agree.” The total score ranged from 9 to 45, and mean scores were categorized into three levels according to Bloom [19]—low level (15–59), moderate level (60–79), and high level (80–100)—with a higher score indicating higher self-efficacy in preventing COVID-19 infection. The CVI of self-efficacy in preventing COVID-19 infection was 0.88. The Cronbach’s alpha coefficient of the questionnaire was 0.84, indicating good internal consistency reliability.

### 2.8. COVID-19 Preventive Behaviors

The questionnaire on COVID-19 preventive behaviors consisted of 15 items, each falling under one of the following three subscales: (1) strength-building behaviors; (2) compliance with DMHTT measures (D = distancing; M = mask wearing; H = handwashing; T = testing (measuring the temperature and getting tested for COVID-19); and T = Thai Cha Na application check, which allows users to register themselves while approaching areas, places, or buildings at risk of COVID-19 infection, access to personal travel information; and (3) screening and vaccinations. Each item was rated based on a 5-point scale: 1 = “not practiced” and 5 = “practiced often”. The total score ranged from 15 to 75, and mean scores were categorized into three levels according to Bloom [19]—low level (15–59), moderate level (60–79), and high level (80–100)—with a higher score indicating higher COVID-19 preventive behaviors in preventing COVID-19 infection. The CVI of COVID-19 preventive behaviors in preventing COVID-19 infection was 0.93. The Cronbach’s alpha coefficient of the questionnaire was 0.79, indicating good internal consistency reliability.

### 2.9. Sociodemographic Variables

The general questions were developed by the PI and numbered eight in all: demographic characteristics (sex, age, education level, marital status, occupation, income), family structure, and chronic illnesses.

### 2.10. Statistical Analysis

The data were analyzed using the IBM^®^ SPSS^®^ 25.0 software (IBM: Armonk, NY, USA). Descriptive statistics were used to analyze the participants’ sociodemographic data. The associations of age, income, knowledge about COVID-19, perceived COVID-19 risk, perceived COVID-19 severity, COVID-19 response efficacy, and self-efficacy in preventing COVID-19 infection with COVID-19 preventive behaviors were examined using Pearson’s product-moment correlation coefficient. We used multiple regression analysis to determine the ability of each variable to predict the COVID-19 preventive behaviors of the older adults in the study. All the assumptions of multivariate normality were met. All analyses were performed with a significance level α = 0.05.

## 3. Results

### 3.1. Characteristics of the Participants

The questionnaire was distributed among 400 older adults (response rate of 100%). Of the 400 participants in the current study, 57.0% (*n* = 227) were female, 252 (63.0%) were aged 60–64 years, 45.0% (*n* = 180) completed the primary education level, and 24.3% (*n* = 97) completed the secondary school level. Most of the participants (61.8%; *n* = 247) were currently married. Regarding occupation, most of the participants (33.5%; *n* = 194) were vendors/private business owners, and 30.7% (*n* = 123) were employees, while 7.25% (*n* = 29) were retired. The incomes were generally uniformly spread between 143 US dollars and 286 US dollars per month (47.5%; *n* = 190). Most of the participants (61.8%; *n* = 247) were living with their nuclear families, while 23.0% (*n* = 92) were living with their extended families. Most reported having chronic illnesses (62.5%; *n* = 250), while 37.5% (*n* = 150) reported having no chronic illnesses.

### 3.2. Knowledge about COVID-19

As shown in Table 1, most of the participants (88.3%; *n* = 353) had a high level of knowledge about COVID-19, followed by a moderate level of knowledge (11.7).

### 3.3. Characteristics of Protection Motivation

As shown in Table 1, of the 400 participants in the current study, 81.5% (*n* = 326) had a high level of overall perceived COVID-19 risk, 18.5% (*n* = 74) had a moderate level; 88.8% (*n* = 355) had a high level of overall perceived COVID-19 severity, 11.2% (*n* = 45) had a moderate level; 64.2% (*n* = 257) had a high level of overall COVID-19 response efficacy, and 35.8% (*n* = 143) had a moderate level; 58.8% (*n* = 235) had a high level of self-efficacy in preventing COVID-19 infection, 40.8% (*n* = 163) had a moderate level, and 0.4% (*n* = 2) had a low level.

### 3.4. COVID-19 Preventive Behavior Level

As shown in Table 1, most of the study participants had a high level of overall COVID-19 preventive behaviors (70.8%). When preventive behaviors in separate areas were considered, most of the older adults in the current study were found to have a high level of strength-building behaviors (48.4%), followed by a moderate level (40.8%) and a low level (10.8%). In the area of compliance with the DMHTT measures, most of the participants had high compliance (86.0%), followed by moderate compliance (14.0%). Most of the participants had high compliance in screening and vaccinations (96.0%), followed by moderate compliance (3.7%) and low compliance (0.3%).

As shown in Table 2, knowledge about COVID-19, perceived COVID-19 risk, perceived COVID-19 severity, COVID-19 response efficacy, and self-efficacy in preventing COVID-19 infection were found to be correlated with the COVID-19 preventive behaviors of the study participants (*p* < 0.05). Age and income were found to be uncorrelated (*p* > 0.05).

The factors that could predict COVID-19 preventive behaviors were analyzed in the current study through stepwise multiple regression. It was found that COVID-19 response efficacy, self-efficacy in preventing COVID-19 infection, knowledge about COVID-19, and gender could predict the COVID-19 preventive behaviors of older adults in Bangkok, Thailand, accounting for approximately 14.3% of the variance in such COVID-19 preventive behaviors. As shown in Table 3, among the four aforementioned factors, self-efficacy in preventing COVID-19 infection had the highest influence on the COVID-19 preventive behaviors of the study participants (β = 0.224), followed by COVID-19 response efficacy (β = 0.171), knowledge about COVID-19 (β = 0.110), and gender (β = −0.102).

## 4. Discussion

In the current study, we focused on older adults (60–69 years) because many of them continue to perform activities in person, travel for work, and socialize with others; therefore, they should be aware of COVID-19. It was found in this study that the average incomes of participants ranged from 143 US dollars to 286 US dollars per month, which is lower than the mean income in Thailand [20]. This is because the participants of this study were older adults, and only 30.7% of participants were employees, while 7.25% were retired. We also found that 61.8% of the participants lived with their nuclear families, while 23.0% lived with their extended families. This could be because the structure of family in Thailand has changed from extended to nuclear family (living alone), which might impact the social support available to assist in self-care [21], especially in older adults.

We found that most of the study participants had a high-level of overall COVID-19 preventive behaviors (70.8%). This can be attributed to the campaigns for the adoption of self-protection behaviors against COVID-19, and the dissemination of the guidelines of the government measures against COVID-19 in Thailand, which were practiced by older adults until the measures became habitual practices in their daily lives [9,13]. In addition, the emphasis placed on disease severity and the effects of COVID-19 infection on one’s health and life, encouraged older adults to adopt appropriate disease prevention behaviors [22,23]. When COVID-19 preventive behaviors were considered separately, those in screening and vaccinations were found to be the highest (96.0%). This was because older people had to comply with the Ministry of Public Health measures, and the Thai government had policies regarding the provision of vaccines for every person in Thailand free of charge, based on human rights, ethics, equality, and consent [9]. Furthermore, vaccinations were an important tool in managing COVID-19, including reducing the cases of severe illness and death, as evidenced by the fact that 76.0% of the study participants had been injected with a COVID-19 vaccine, and that 73.8% intended to be vaccinated against COVID-19 when notified by the public health officials in the places where they were registered. Most of the participants (86.0%) had high compliance with the DMHTT measures, complying with such measures regularly and accepting disease prevention practices as part of their daily lives [24,25].

We also found that the factors associated with COVID-19 preventive behaviors were gender, knowledge about COVID-19, perceived COVID-19 risk, perceived COVID-19 severity, COVID-19 response efficacy, and self-efficacy in preventing COVID-19 infection. These findings are important for developing guidelines to effectively combat COVID-19. Self-efficacy in preventing COVID-19 infection, COVID-19 response efficacy, knowledge about COVID-19, and gender were able to predict COVID-19 preventive behaviors, accounting for approximately 14.3% of the variance therein. Self-efficacy in preventing COVID-19 infection showed the strongest influence on the study participants’ COVID-19 preventive behaviors. Most of the participants (57.8%) believed that people should always wear a mask when in a public place, and 49.8% believed that maintaining a distance of 1–2 m from others could reduce infections and the spread of COVID-19. The results of our study are consistent with those of previous studies in which perceived self-efficacy in preventing COVID-19 infection was found to be the factor with the greatest influence on behaviors that could prevent COVID-19 infection among older adults [8,26]. In addition, previous researchers found that perceived self-efficacy in preventing COVID-19 infection was significantly positively correlated with COVID-19 preventive behaviors [27,28].

Our study also revealed that COVID-19 response efficacy has a great influence on the COVID-19 preventive behaviors of older adults. We found that the older adults in the current study had a high level of COVID-19 response efficacy, with 56.8% of them believing that frequently washing their hands with soap or alcohol gel could prevent COVID-19 infection, and 54.3% believing that screening would help identify infected individuals, that infected people should be isolated from non-infected ones to prevent the spread of COVID-19, and to provide appropriate care for the former. In addition, previous studies found that the effectiveness of COVID-19 responses was significantly positively correlated with COVID-19 preventive behaviors [29,30].

Another factor that was found to influence the COVID-19 preventive behaviors of the older adults in the current study was knowledge about COVID-19. We found that the older adults in the current study had a high level of knowledge about COVID-19. Good preventive behaviors are likely to be influenced by older adults’ knowledge about and attitudes toward COVID-19 [9]. Thus, knowledge about COVID-19, especially among older adults, is essential in battling the COVID-19 pandemic [11,31]. Previous studies have also found that knowledge about COVID-19 was positively correlated with COVID-19 preventive behaviors [13,32].

Gender was also found to influence the COVID-19 preventive behaviors of the older adults in the current study. We found that the females in the current study had high-levels of COVID-19 preventive behaviors. The results of our study are consistent with those of previous studies in which the male and female participants were found to have had significantly different preventive behaviors [33,34]. This is also consistent with previous studies [29,35], including two studies conducted in Israel and Iran where most of the female participants were found to have had higher levels of preventive behaviors than the male participants. Interestingly, protective motivations, perceived COVID-19 risk, and perceived COVID-19 severity were not able to predict the COVID-19 preventive behaviors of the older adults in the current study. The results of the study suggest that the protection motivation theory provides an appropriate theoretical framework and useful insights for better understanding people’s motivations for adopting behavioral modifications during the current pandemic. The results of our study and its inconsistency with national survey studies in the United States found that perceived COVID-19 risk, and perceived COVID-19 severity, were associated with COVID-19 preventive behaviors [36,37]. However, we believe that the protection motivation theory provides an appropriate theoretical framework and useful insights for better understanding people’s motivations for adopting behavioral modifications [38] during the current pandemic. However, these cross-sectional data do not allow us to draw a causal conclusion; future research is required to confirm our findings in other multiple variables or factors (e.g., health literacy, resilience, emotions) with larger sample sizes.

The current study has some limitations that should be addressed in future studies. First, as the current study used a cross-sectional survey, conclusions based on its findings may not be as definitive as those based on the findings of an experimental or quasi-experimental study. Second, we used questionnaires by self-assessed behavior with good content validity to assess COVID-19 preventive behaviors of older adults; all questionnaires were completed anonymously, but social desirability and potentially biased ratings of self-assessed behavior cannot be ruled out. Future studies should use other approaches to describe this phenomenon and it is important to consider reducing potential bias. Third, our findings may not be generalizable to other settings because they were based on a single province of Thailand. Fourth, convenience sampling was used in the current study, and all the participants gave their consent to participate in the study, which could indicate selection bias. Fifth, all the participants could somehow communicate in Thai. Therefore, it may not be possible to apply the findings of this study to those who cannot communicate in Thai. Finally, we examined factors associated with COVID-19 preventive behaviors of older people aged 60–69; however, having a cognitive impairment may have affected our findings, which introduces potential bias. Therefore, future studies should screen for cognitive impairment in older adults that may influence their health outcomes.

## 5. Conclusions

The results of the current study indicate that higher self-efficacy in preventing COVID-19 infection, higher COVID-19 response efficacy, and higher knowledge about COVID-19 were related to higher COVID-19 preventive behaviors. We also found that females are related to higher COVID-19 preventive behaviors of the older adults in Bangkok, Thailand. The present findings may be applied to policymaking to establish appropriate interventions for promoting good COVID-19 preventive behaviors to prevent further COVID-19 infection and spread, particularly among older people. However, other research methods and more predictors need to be observed in future research.

## Figures and Tables

**Table 1:** Knowledge about COVID-19, protection motivation, and COVID-19 preventive behaviors of the study participants.

Factor	Number	Percentage
Knowledge about COVID-19		
Low	0	0.0
Moderate	47	11.7
High	353	88.3
Perceived COVID-19 risk		
Low	0	0.0
Moderate	74	18.5
High	326	51.5
Perceived COVID-19 severity		
Low	0	0.0
Moderate	45	11.2
High	355	88.8
COVID-19 response efficacy	0	0.0
Low	0	0.0
Moderate	143	35.8
High	257	64.2
Self-efficacy in preventing COVID-19 infection		
Low	2	0.4
Moderate	163	40.8
High	235	58.8
Overall COVID-19 preventive behaviors		
Low	0	0.0
Moderate	117	29.2
High	283	70.8
Strength-building behaviors		
Low	43	10.8
Moderate	163	40.8
High	194	48.4
Compliance with DMHTT measures		
Low	0	0.0
Moderate	56	14.0
High	344	86.0
Screening and vaccinations		
Low	1	0.3
Moderate	15	3.7
High	384	96.0

Factor correlations with COVID-19 preventive behaviors.

**Table 2:** Factor correlations with COVID-19 preventive behaviors.

Variable	Coefficient Correlation (r)	*p*-Value
Age	0.012	0.81
Income	−0.031	0.53
Knowledge about COVID-19	0.107	0.03
Perceived COVID-19 risk	0.104	0.03
Perceived COVID-19 severity	0.171	<0.001
COVID-19 response efficacy	0.312	<0.001
Self-efficacy in preventing COVID-19 infection	0.340	<0.001

Influence of COVID-19 response efficacy, self-efficacy in preventing COVID-19 infection, knowledge about COVID-19, and gender on COVID-19 preventive behaviors.

**Table 3:** Results of the stepwise multiple regression analysis of the factors with an influence on and ability to predict the COVID-19 preventive behaviors of the study participants.

Variable	B	Beta	*t*	*p*-Value
Self-efficacy in preventing COVID-19 infection	0.203	0.224	3.731	<0.001
COVID-19 response efficacy	0.163	0.171	2.852	<0.05
Knowledge about COVID-19	0.155	0.110	2.237	0.01
Gender (female vs. male *)	−0.093	−0.102	−2.186	0.02

Note: * reference group.

## Data Availability

The datasets generated during and/or analyzed, and questionnaires during the current study are available from the corresponding author upon reasonable request.
